# Integrin Signaling Imbalance in Periodontitis: A Stage-Dependent Link Between Inflammation, Bone Resorption and Regenerative Failure

**DOI:** 10.3390/biom16070967

**Published:** 2026-06-30

**Authors:** Fredy Mardiyantoro, Meircurius Dwi Condro Surboyo, Andari Sarasati, Tetsuya Matsuguchi

**Affiliations:** 1 Department of Oral and Maxillofacial Surgery, Faculty of Dentistry, Universitas Brawijaya, Malang 65145, Indonesia; tmatsugu@dent.kagoshima-u.ac.jp; 2 Department of Oral Medicine, Faculty of Dental Medicine, Universitas Airlangga, Surabaya 60132, Indonesia; andari.sarasati@fkg.unair.ac.id; 3 Department of Oral Biochemistry, Field of Developmental Medicine, Graduate School of Medical and Dental Sciences, Kagoshima University, Kagoshima 890-8544, Japan

**Keywords:** integrins, periodontitis, bone remodeling, inflammation

## Abstract

Periodontitis is a chronic inflammatory disease driven largely by dysregulated host responses that lead to destruction of periodontal tissues. Integrins are heterodimeric transmembrane receptors that regulate cell adhesion and bidirectional signaling in epithelial cells, immune cells, periodontal ligament fibroblasts, and osteoclasts. During disease progression, integrin-related responses may shift across overlapping molecular phases. Epithelial integrins such as α3β1 and α6β4 support barrier integrity, whereas α5β1 may facilitate microbial interaction and inflammatory signaling. β2 integrins and α4β1 contribute to leukocyte recruitment and inflammatory amplification, whereas increased α9β1-associated signaling and reduced αvβ6-mediated regulation of transforming growth factor β (TGF-β) may promote inflammatory persistence. Matrix-associated integrins, including α2β1 and α11β1, support extracellular matrix (ECM) organization and mechanotransduction, whereas αvβ3 cooperates with Receptor activator of nuclear factor kappa B ligand (RANKL) to promote osteoclast activity and alveolar bone resorption. Impaired β1 integrin-dependent signaling and potentially reduced αvβ5-associated efferocytosis may contribute to defective resolution and regeneration. Importantly, integrin expression, activation, and downstream signaling are distinct, and the strength of evidence varies among integrin subtypes. This review proposes a conceptual framework in which periodontitis reflects a dynamic imbalance in integrin-mediated processes that link inflammation, bone resorption, and regenerative failure, rather than being a direct equivalent of clinical periodontal stages or grades.

## 1. Introduction

Periodontitis is a chronic inflammatory disease characterized by progressive destruction of the tooth-supporting tissues, including gingiva, periodontal ligament (PDL), cementum, and alveolar bone [1,2]. Although initiated by dysbiosis-associated biofilms, the severity and progression of tissue breakdown are largely determined by the host response rather than solely by bacterial infection [3,4,5]. Growing evidence supports the concept that periodontitis represents a failure of immune regulation and tissue homeostasis, in which persistent inflammatory signaling becomes coupled to pathological bone remodeling [6,7,8]. This underscores the need for therapeutic strategies directed toward the dominant molecular imbalance in the periodontal microenvironment [9,10,11].

Integrins are transmembrane heterodimeric receptors composed of α and β subunits that mediate cell–cell and cell–ECM interactions. Beyond their traditional role as adhesion molecules, integrins are currently recognized as bidirectional signaling receptors capable of transmitting both outside-in and inside-out signals [12]. Through the activation of intracellular pathways such as focal adhesion kinase (FAK), mitogen-activated protein kinases (MAPK), nuclear factor kappa B (NF-κB), phosphoinositide 3-kinase (PI3K)/Akt, and Rho-family GTPases, integrins regulate cellular migration, survival, proliferation, differentiation, and cytokine production [13]. In periodontal tissues, these functions play essential roles in immune cell recruitment, epithelial barrier integrity, osteoclast activation, and matrix remodeling [14,15].

Multiple integrin subtypes contribute to distinct but interconnected biological processes during periodontal disease progression. Epithelial-associated integrins, including α3β1 and α6β4, maintain barrier integrity and attachment to the basement membrane [16], whereas α5β1 mediates ECM adhesion and can facilitate bacterial internalization and pro-inflammatory signaling [17]. Leukocyte-associated integrins, particularly β2 family members such as LFA-1 and Mac-1, together with α4β1, regulate immune cell recruitment and activation, while additional integrins, including αXβ2 and αDβ2, contribute to antigen presentation and the persistence of inflammatory responses [18]. Integrins associated with inflammatory progression, such as α9β1, sustain signaling pathways that promote chronic inflammation [19]. Collagen-binding integrins, including α2β1 and α11β1, regulate ECM organization and mechano-transduction, while αvβ3 plays a central role in osteoclast adhesion and bone resorption, linking inflammatory signaling to alveolar bone loss [15]. In contrast, αvβ6 expressed in junctional epithelium activates latent transforming growth factor-β (TGF-β) and functions as a local suppressor of inflammation [20].

Despite growing evidence of integrin involvement, many studies have focused primarily on expression patterns rather than on the mechanisms by which integrin-mediated signaling links inflammation, bone resorption, and regenerative failure in periodontal disease [21,22]. A comprehensive mechanistic understanding of these pathways is required to determine the drivers of periodontal tissue destruction, the amplification of osteoclastogenesis, and the compromised outcomes of regenerative therapies in inflammatory environments.

In this review, the term “stage-dependent” refers to a conceptual molecular and pathobiological framework rather than to the clinically recognized stages I–IV or grades A–C of periodontitis. The proposed framework describes sequential but overlapping changes in integrin-mediated responses during disease pathogenesis, including epithelial barrier disruption, immune cell recruitment and inflammatory amplification, loss of regulatory signaling, extracellular matrix destruction, osteoclast-mediated bone resorption, and impaired inflammatory resolution and tissue regeneration. These processes are defined primarily by changes in inflammatory status, cellular behavior, and tissue-destructive mechanisms. They should not be interpreted as rigid histopathological phases or direct equivalents of clinical disease severity because several may occur simultaneously within the same periodontal lesion.

Accordingly, this review synthesizes current evidence on integrin-mediated signaling in periodontitis. It explains how changes in integrin expression, activation, and downstream signaling may contribute to epithelial barrier dysfunction, inflammatory amplification, RANKL-dependent bone resorption, and impaired periodontal regeneration. By integrating evidence from host–pathogen interactions, inflammatory signaling, osteoclastogenesis, extracellular matrix remodeling, and mechanotransduction, we propose that periodontitis involves a progressive imbalance in integrin signaling networks. This framework may support the identification of molecularly informed therapeutic targets and guide future studies examining the relationship between integrin signaling and clinical disease characteristics.

## 2. Integrins: Structure, Distribution, and Physiological Function

Integrins are heterodimeric transmembrane receptors composed of an α and a β subunit. In humans, the 18 α and 8 β subunits combine to form 24 distinct integrin receptors. These receptors function as adhesion molecules that connect cells to the ECM and to other cells [13,23]. However, integrins are not merely structural anchors. They are dynamic signaling receptors that regulate cellular behavior through bidirectional communication across the plasma membrane [13,15].

### 2.1. α Subunit Structure and Functional Classification

The α subunit primarily determines ligand specificity. Structurally, it consists of a large extracellular domain, a single-pass transmembrane segment, and a short cytoplasmic tail. The extracellular portion forms a β-propeller structure that binds ECM proteins such as collagen, fibronectin, laminin, and vitronectin. In selected α subunits, including αL and αM, an inserted I-domain enhances ligand-binding capacity, particularly in leukocyte integrins such as LFA-1 (αLβ2) and Mac-1 (αMβ2) [12,24,25].

The cytoplasmic tail of the α subunit is relatively short and does not possess intrinsic catalytic activity. However, it participates in inside-out signaling by contributing to conformational regulation and cytoskeletal linkage [25,26].

Functionally, α subunits can be categorized based on their ligand-binding properties, which determine their biological roles in periodontal tissues (Table 1). This classification reflects how integrins interact with ECM components, immune ligands, and regulatory molecules to coordinate tissue homeostasis and inflammatory responses.

### 2.2. β Subunit Structure and Functional Classification

The β subunit plays a dominant role in intracellular signaling. It contains a large extracellular region, a transmembrane helix, and a cytoplasmic tail with conserved motifs that bind adaptor proteins such as talin and kindlin. These interactions are essential for integrin activation, clustering, and connection to the actin cytoskeleton [12,25,27].

In periodontal biology, integrins are most commonly classified by their β subunit family, as this often predicts cellular distribution and biological function (Table 2).

This classification provides a simplified conceptual framework. The β1 integrins, such as α5β1 and α11β1, are widely expressed in PDL fibroblasts and epithelial cells, where they regulate ECM organization and mechano-transduction [15,28]. The β2 integrins, including LFA-1 and Mac-1, are restricted to leukocytes and are essential for immune cell adhesion and transmigration [27,29]. The β3 integrins, particularly αvβ3, are highly expressed on osteoclasts and are indispensable for bone resorptive activity [30]. The β6 integrin, which pairs exclusively with αv (forming αvβ6), is primarily expressed in epithelial tissues and plays a regulatory role in inflammation by activating latent TGF-β [13].

**Table 1:** Functional classification of α-subunit integrins in periodontal tissues.

α Subunit Group	Representative Integrins	Major Ligands	Main Functional Role	References
Collagen-binding	α2β1 α11β1	Collagen	ECM organization Collagen remodeling Mechano-transduction	[31,32,33,34,35]
Fibronectin-binding	α5β1	Fibronectin	Matrix adhesion Bacterial internalization Inflammatory signaling	[33,36,37]
Laminin-binding	α3β1 α6β4	Laminin	Epithelial adhesion Basement membrane stability	[38,39]
Leukocyte-specific (I-domain)	αLβ2 (LFA-1) αMβ2 (Mac-1) αXβ2 αDβ2	ICAMs Complement proteins	Leukocyte adhesion, migration and immune activation	[40,41]
αv integrins (RGD-binding)	αvβ3 αvβ5 αvβ6	Vitronectin Fibronectin Latent TGF-β	Bone resorption Immune regulation Resolution of inflammation	[42,43,44,45,46,47,48]
Inflammation- associated	α9β1	Osteopontin Tenascin-C	MAPK-associated signaling and possible inflammatory persistence	[19]

**Table 2:** β family integrin and its function.

β Family	Representative Integrins	Main Cell Type	Main Role
β1	α2β1 α3β1 α5β1 α4β1 α9β1 α11β1	Fibroblasts Epithelium	Matrix adhesion Mechano-transduction Regeneration
β2	αLβ2 (LFA-1) αMβ2 (Mac-1) αXβ2 αDβ2	Leukocytes	Immune trafficking Adhesion Inflammatory activation
β3	αvβ3	Osteoclasts	Bone resorption
β5	αvβ5	Macrophages Epithelium	Efferocytosis Resolution of inflammation
β6	αvβ6	Epithelial cells	Anti-inflammatory regulation (TGF-β activation)

### 2.3. Integrin Expression, Conformational Activation, and Signaling

**Figure 1:**
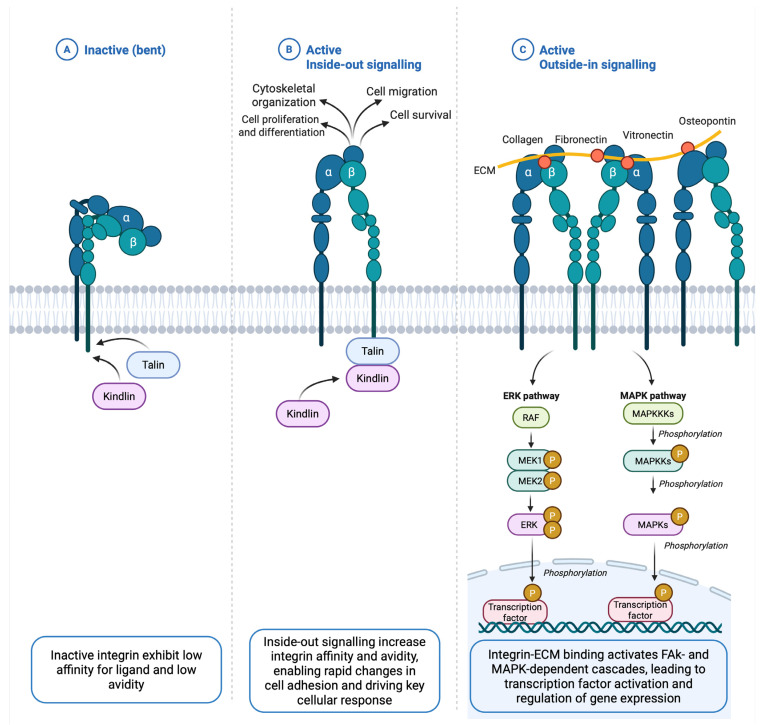
Integrin conformational activation and bidirectional signaling. (**A**) In the inactive state, integrins adopt a bent conformation with low ligand affinity and avidity. (**B**) During inside-out signaling, intracellular adaptor proteins such as talin and kindlin bind to the β cytoplasmic tail, promoting integrin extension and increasing affinity and avidity for extracellular ligands. This activation enables rapid changes in cell adhesion and supports cytoskeletal organization, cell migration, survival, proliferation, and differentiation. (**C**) During outside-in signaling, activated integrins bind ECM ligands, including collagen, fibronectin, vitronectin, and osteopontin. Ligand engagement promotes intracellular signaling through FAK-dependent pathways, including Src and PI3K/Akt, and MAPK/ERK-dependent cascades, leading to phosphorylation of downstream targets, activation of transcription factors, and regulation of gene expression. The figure illustrates changes in integrin conformational and signaling states and does not imply a change in receptor expression or abundance (www.biorender.com).

Integrin expression, activation, and downstream signaling represent distinct levels of regulation. Expression refers to receptor abundance at the mRNA, total protein, cell-surface, or tissue-localization levels and does not necessarily indicate functional activity. Integrins exist in inactive, bent conformations with low ligand affinity and avidity and in active, extended conformations capable of effective ligand binding. During inside-out signaling, adaptor proteins such as talin and kindlin bind to the β-subunit cytoplasmic tail, promoting conformational rearrangement, receptor clustering, cytoskeletal coupling, and increased ligand affinity or avidity [49]. Subsequent ligand engagement initiates outside-in signaling through FAK-dependent pathways involving Src-family kinases, Pyk2, PI3K/Akt, and MAPK cascades, including ERK signaling [15]. These pathways regulate phosphorylation, transcription-factor activity, cytoskeletal organization, migration, survival, proliferation, and differentiation [13,50] (Figure 1). Throughout this review, “increased” or “decreased expression” refers specifically to changes in receptor abundance, whereas “activation,” “engagement,” and “signaling” are used only when supported by functional or mechanistic evidence.

Cooperation between the α and β subunits determines ligand specificity, cellular distribution, and signaling capacity. In periodontal tissues, these properties enable integrins to regulate immune responses, bone remodeling, and the integrity of epithelial and connective tissues [6,15,20,22,28]. During periodontitis, changes in integrin expression, activation, or downstream signaling may shift these functions from maintaining tissue homeostasis toward chronic inflammation, alveolar bone destruction, and impaired repair [6,51].

## 3. Integrins in Pathogenesis of Periodontitis

**Figure 2:**
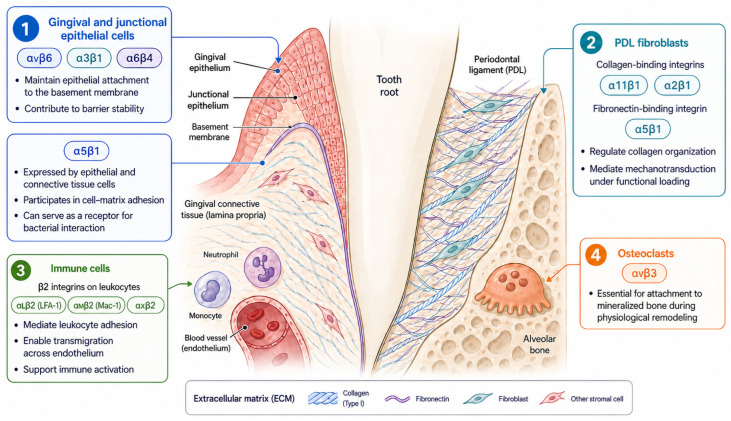
Distribution and functional roles of integrins in periodontal tissues. Periodontal epithelial cells, PDL fibroblasts, immune cells, and osteoclasts express the major integrin subtypes. (1) Junctional and gingival epithelial cells express αvβ6, α3β1, and α6β4, which support epithelial attachment to the basement membrane and barrier stability. α5β1 is expressed by epithelial and connective tissue cells and contributes to cell–matrix adhesion and bacterial interactions. (2) PDL fibroblasts express collagen-binding integrins α11β1 and α2β1, together with fibronectin-binding α5β1, which regulate collagen organization and mechano-transduction under functional loading. (3) Immune cells, including neutrophils and monocytes, express β2 integrins such as αLβ2, αMβ2, and αXβ2, which mediate leukocyte adhesion, transmigration, and immune activation. (4) Osteoclasts strongly express αvβ3, which is essential for attachment to mineralized bone during physiological remodeling (www.biorender.com).

Integrins are widely distributed across periodontal tissues, where they coordinate epithelial integrity, immune surveillance, ECM organization, and bone remodeling [15]. Junctional and gingival epithelial cells express integrins such as αvβ6, α3β1, and α6β4, which maintain epithelial attachment to the basement membrane and contribute to barrier stability [52,53]. In addition, α5β1, expressed by epithelial and connective tissue cells, participates in cell–matrix adhesion and can serve as a receptor for bacterial interactions. PDL fibroblasts express collagen-binding integrins, including α11β1 and α2β1, as well as fibronectin-binding α5β1, which regulate collagen organization and mechano-transduction under functional loading [28]. Immune cells, particularly neutrophils and monocytes, express β2 integrins including αLβ2 (LFA-1), αMβ2 (Mac-1), and αXβ2, which mediate leukocyte adhesion, transmigration, and immune activation [27,29]. Osteoclasts strongly express αvβ3 integrin, which is essential for attachment to mineralized bone during physiological remodeling [54] (Figure 2).

**Figure 3:**
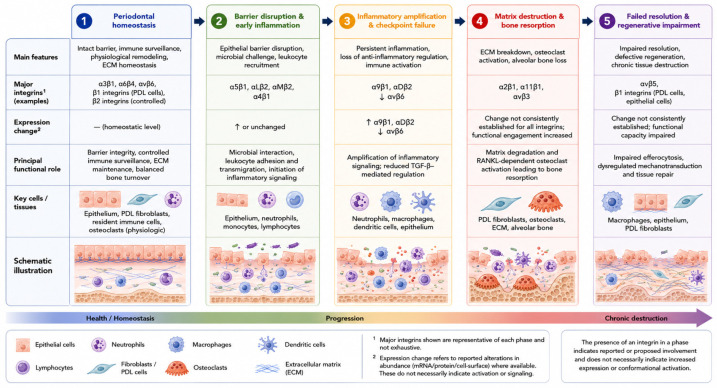
Proposed molecular progression of integrin dysregulation in periodontitis. The figure summarizes sequential yet overlapping molecular and pathobiological phases that extend from periodontal homeostasis to barrier disruption, inflammatory amplification, matrix destruction, bone resorption, and failed resolution or regeneration. (1) During homeostasis, epithelial, leukocyte, and PDL integrins support barrier stability, controlled immune surveillance, ECM organization, and physiological remodeling. (2) Early microbial challenge is associated with epithelial–microbial interactions and β2 integrin-dependent leukocyte recruitment. (3) Persistent inflammation is accompanied by α9β1- and αDβ2-associated inflammatory persistence and reduced αvβ6-mediated regulatory capacity. (4) Matrix-associated integrins and osteoclast αvβ3 subsequently contribute to connective tissue disruption and bone resorption. (5) Impaired β1 integrin-dependent mechanotransduction and potentially αvβ5-associated efferocytosis may contribute to failed resolution and regeneration. These phases represent a conceptual molecular framework and do not correspond directly to the clinically recognized stages or grades of periodontitis. The presence of an integrin within a phase indicates reported or proposed involvement and does not necessarily indicate altered expression or conformational activation (www.biorender.com).

Under homeostatic conditions, these integrins function as mechanochemical sensors that preserve tissue equilibrium by coordinating epithelial stability, controlled immune trafficking, balanced bone turnover, and adaptive responses to mechanical stress [15,20,28,52]. Integrins such as α3β1 and α6β4 maintain epithelial barrier integrity [38], while αvβ6 contributes to local immune regulation [53]. β2 integrins regulate transient leukocyte recruitment required for immune surveillance [55], and β1 integrins in PDL fibroblasts support ECM organization and mechanical adaptation [20]. In periodontitis, the transition from health to disease may involve changes at several distinct regulatory levels, including integrin expression and localization, conformational or ligand-dependent activation, and downstream signaling. These changes should be distinguished because altered receptor abundance does not necessarily indicate altered functional activity (Figure 3).

At the epithelial interface, disruption of integrin-mediated attachment represents an early event in disease initiation. Based on their established roles in epithelial adhesion, changes in the expression, localization, or organization of α3β1 and α6β4 may compromise epithelial attachment and barrier function [20,38]. However, these observations do not necessarily demonstrate altered integrin conformational activation. Concurrently, α5β1 integrin serves as a binding and entry receptor for periodontal pathogens such as *Porphyromonas gingivalis*, enabling bacterial internalization and activation of downstream inflammatory signaling pathways [37]. These interactions highlight the role of integrins as interfaces between microbial challenge and host cell signaling.

The microbial–integrin interface may extend beyond *Porphyromonas gingivalis*. *Aggregatibacter actinomycetemcomitans* outer-membrane components have been reported to stimulate epithelial responses through fibronectin-associated integrin pathways, suggesting that ECM proteins can serve as molecular bridges between bacterial components and host receptors [56]. *Fusobacterium nucleatum* also binds fibronectin associated with gingival epithelial surfaces [57], whereas *Treponema denticola* interacts with several ECM proteins, including fibronectin, laminin, fibrinogen, and collagen [58]. These matrix-binding properties could facilitate indirect interaction with fibronectin- or laminin-binding integrins and thereby influence bacterial adhesion, epithelial signaling, or tissue invasion [17]. However, direct engagement of specific integrin heterodimers has not been consistently demonstrated in these organisms. These mechanisms should therefore be considered plausible ECM-mediated interactions rather than established direct integrin-dependent pathways. Polymicrobial biofilms may also indirectly alter integrin abundance, as mixed biofilms containing *Porphyromonas gingivalis* and *Fusobacterium nucleatum* have been reported to reduce epithelial αvβ6 expression, potentially weakening local TGF-β-mediated regulatory control [59].

β2 integrins largely mediate the subsequent immune response. LFA-1 and Mac-1 play central roles in leukocyte adhesion and transmigration, allowing neutrophils and monocytes to accumulate at sites of infection [55]. Additional integrins, such as α4β1, contribute to the recruitment of monocytes and lymphocytes. In contrast, αXβ2 and αDβ2 may contribute to antigen presentation and inflammatory cell retention, based primarily on their established functions in other inflammatory contexts [60], although direct periodontal evidence remains limited. While this recruitment is essential for microbial control, sustained β2 integrin-dependent adhesion and signaling may amplify reactive oxygen species production, protease release, and pro-inflammatory cytokine signaling, contributing to collateral connective tissue damage [61]. Conversely, defective β2 integrin function, as observed in leukocyte adhesion deficiency, results in impaired neutrophil recruitment and severe early-onset periodontitis [62], underscoring the need for tight regulation of these pathways.

Although these early immune responses are essential for microbial control, their persistence and failure to resolve can transform a protective host-defense response into chronic inflammatory dysregulation. As inflammation progresses, additional integrins may contribute to inflammatory persistence. Increased α9β1 expression has been reported in inflamed periodontal tissues. Functional studies in periodontal and related inflammatory models suggest that α9β1 engagement may enhance MAPK-related signaling and support inflammatory cell survival. However, direct evidence that α9β1 prolongs neutrophil survival in human periodontal lesions remains limited [63]. Similarly, αDβ2 may contribute to macrophage retention and chronic inflammatory cell accumulation, but this mechanism is inferred primarily from non-periodontal inflammatory models. These pathways should therefore be regarded as plausible contributors to chronicity rather than as conclusively established periodontal mechanisms.

At the same time, reduced αvβ6 expression in the junctional or pocket epithelium may decrease the local capacity to activate latent TGF-β, thereby weakening an important anti-inflammatory checkpoint [13,64]. Functional studies using epithelial cells and integrin-deficient animal models support the regulatory role of the αvβ6–TGF-β axis. Nevertheless, receptor abundance and conformational activation remain distinct parameters, and reduced expression should not automatically be interpreted as direct evidence of reduced receptor activation.

Chronic inflammation also disrupts integrin-mediated ECM regulation. Collagen-binding integrins such as α2β1 and α11β1 normally contribute to PDL structure and matrix organization. Chronic inflammation may disturb α2β1- and α11β1-associated matrix signaling; however, current evidence more directly supports their physiological roles in collagen organization and PDL function than their specific dysregulation during periodontitis [31,34]. Such disruption may contribute to soft tissue breakdown and loss of structural integrity within periodontal tissues. In addition, β1 integrin-dependent mechano-transduction becomes dysregulated in PDL fibroblasts and progenitor cells, impairing osteogenic differentiation and limiting the tissue’s regenerative capacity [65].

Alveolar bone destruction is a major consequence of dysregulated integrin-mediated signaling. Osteoclast-associated αvβ3 mediates adhesion to bone-matrix proteins and activates intracellular mediators such as c-Src and Pyk2, promoting actin-ring formation, cytoskeletal polarization, and development of the resorptive apparatus [54]. Inflammatory cytokines increase RANKL expression, while αvβ3 engagement cooperates with RANK signaling to enhance osteoclast maturation and bone-resorptive activity [6,54]. Thus, αvβ3 provides a functional link between inflammatory signaling and structural bone loss. However, direct human evidence correlating αvβ3 activation with the severity or progression of alveolar bone destruction remains limited.

Impaired clearance of apoptotic cells may further delay inflammatory resolution. αvβ5 has been implicated in efferocytosis in other inflammatory settings and may contribute to apoptotic-cell clearance in periodontal tissues [66]. However, its direct role in periodontitis has not been established. Accordingly, αvβ5-associated efferocytosis should be considered a plausible resolution mechanism requiring direct periodontal validation rather than a confirmed pathway.

Collectively, integrins function as interconnected signaling hubs that link epithelial barrier integrity, microbial interaction, immune cell recruitment, inflammatory amplification, ECM remodeling, osteoclast activation, and tissue repair. The evidence supporting these roles varies substantially among integrin subtypes and includes direct periodontal observations, experimental periodontal models, and mechanistic extrapolations from the broader inflammatory literature. The proposed framework, therefore, integrates findings of different levels of directness. It should be interpreted as a working model of periodontal disease progression rather than as a fully established sequence of molecular events (Figure 3).

## 4. Integrin Signaling Network in Periodontal Inflammation and Bone Remodeling

Integrins do not function in isolation. In periodontal tissues, they form interconnected signaling networks that integrate inflammatory cues, microbial stimuli, and mechanical forces [15]. The pathogenic transition from homeostasis to chronic inflammation reflects coordinated activation of integrin-dependent intracellular pathways, particularly FAK, Src family kinases, MAPK cascades, NF-κB signaling, PI3K/Akt, and Rho GTPases [13,67]. In addition to classical integrins such as β2, αvβ3, and αvβ6, other integrins, including α5β1, α9β1, and β1-associated integrins, contribute to this network by linking extracellular interactions with intracellular signaling responses.

### 4.1. Integrin-NF-kB Axis in Inflammatory Amplification

The β2 integrins, including LFA-1 and Mac-1, act as key signaling mediators of leukocyte activation [55]. Upon ligand binding, these integrins cluster and recruit Src kinases and FAK, initiating downstream signaling that converges on NF-κB activation [55]. NF-κB drives transcription of pro-inflammatory cytokines such as IL-1β, TNF-α, and IL-6. While this response is essential for antimicrobial defense, persistent ligand engagement and β2 integrin-dependent signaling sustain NF-κB activation, promoting excessive cytokine production and connective tissue degradation [68].

Additionally, α5β1 integrin, engaged by ECM components or bacterial adhesins from *Porphyromonas gingivalis*, activates JNK and NF-κB pathways [22,37]. This establishes a feedback loop in which bacterial invasion enhances inflammatory signaling, which in turn further modifies integrin activation states. Integrins such as α4β1 may also contribute to NF-κB activation by facilitating recruitment and activation of mononuclear cells within inflamed tissues [55].

### 4.2. Integrin-MAPK Signaling and Chronic Inflammation

Several integrins modulate MAPK pathways, including ERK, JNK, and p38. Increased α9β1 expression has been observed in inflamed periodontal tissues. In contrast, functional studies in periodontal and related inflammatory contexts suggest that α9β1 engagement may enhance MAPK-related signaling and support inflammatory cell survival. However, direct evidence that α9β1 prolongs neutrophil survival in human periodontal lesions remains limited [13]. Likewise, αDβ2 and αXβ2 may contribute to macrophage retention and antigen presentation based mainly on their established functions in other inflammatory settings [65]. These mechanisms should therefore be regarded as plausible contributors to inflammatory persistence rather than as conclusively demonstrated periodontal pathways.

### 4.3. Integrin-RANKL Crosstalk in Osteoclast Activation

The most direct link between inflammation and structural destruction is mediated by αvβ3 integrin on osteoclasts [51,54]. RANKL, produced by activated T cells, fibroblasts, and osteoblast-lineage cells during periodontal inflammation, drives osteoclast differentiation [69]. However, RANKL signaling alone is insufficient for efficient bone resorption.

αvβ3 engagement with bone matrix proteins activates c-Src and Pyk2, promoting cytoskeletal reorganization and actin ring formation. This integrin-dependent signaling synergizes with RANK-mediated activation of NF-κB and NFATc1, amplifying osteoclast maturation and resorptive capacity [70].

Therefore, inflammatory cytokines increase RANKL expression, while αvβ3 integrin ensures structural attachment and functional activation of osteoclasts. This convergence explains how inflammatory signaling leads to alveolar bone loss.

### 4.4. Integrin-TGF-β Axis in Inflammatory Control

In contrast to pro-destructive pathways, αvβ6 integrin, expressed by the junctional epithelium, activates TGF-β-dependent signaling that limits inflammatory responses [13,64]. Reduced αvβ6 expression during periodontitis may decrease the epithelial capacity to activate latent TGF-β. Functional studies using cellular and integrin-deficient animal models support the importance of the αvβ6–TGF-β pathway, although receptor abundance and activation state remain distinct parameters [52,53]. Thus, loss of protective integrin-mediated regulation contributes to inflammatory escalation.

### 4.5. Integrin Signaling and Mechano-Transduction in Regeneration

The β1 integrins, particularly α11β1, α2β1, and α5β1 in PDL fibroblasts, regulate mechanotransduction through the FAK and RhoA/ROCK signaling pathways. These pathways control collagen organization, cytoskeletal tension, and osteogenic differentiation [50,67]. Chronic inflammation disrupts this integrin-dependent mechano-signaling, impairing ECM remodeling and regenerative responses. As a result, regenerative therapies may fail when integrin signaling remains skewed toward inflammatory dominance [71]. In addition, integrins such as αvβ5 may influence resolution by regulating efferocytosis, and their dysfunction may further delay tissue repair.

### 4.6. Integrated Network Perspective

**Figure 4:**
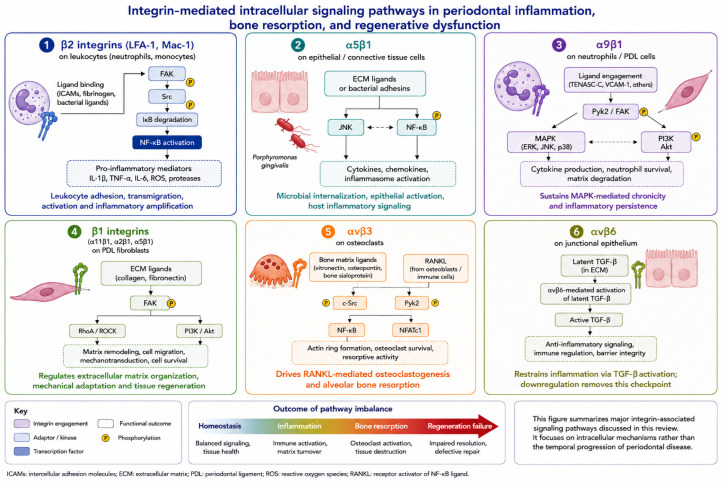
Integrin-associated signaling pathways in periodontal inflammation, bone remodeling, and regeneration. (1) Leukocyte β2 integrins promote FAK/Src-dependent signaling that can converge on NF-κB and inflammatory cytokine production. (2) α5β1 links ECM or microbial interactions with JNK- and NF-κB-associated responses. (3–5) α9β1 may support inflammatory persistence through MAPK and PI3K/Akt-related pathways. (6) In contrast, epithelial αvβ6 contributes to inflammatory regulation by activating latent TGF-β. In osteoclasts, αvβ3 engagement cooperates with RANKL signaling through c-Src, Pyk2, NF-κB, and NFATc1 to support cytoskeletal organization and bone resorption. In PDL cells, β1-family integrins regulate ECM remodeling and mechanotransduction through FAK- and RhoA/ROCK-associated pathways. This figure focuses on signaling mechanisms rather than the temporal progression of periodontal disease (www.biorender.com).

Collectively, β1-, β2-, and αv-containing integrins form an interconnected signaling network in periodontal tissues that coordinates microbial sensing, epithelial regulation, immune cell recruitment, stromal responses, bone remodeling, and tissue repair (Figure 4). These integrin families do not act independently. Instead, they influence one another through shared extracellular ligands, common downstream mediators, and cytokine- and matrix-dependent communication between immune and resident periodontal cells.

During the early phase of periodontal inflammation, α5β1 links microbial stimulation, including interaction with *Porphyromonas gingivalis*, to host inflammatory signaling through JNK- and NF-κB-related pathways [37]. In parallel, αvβ6 expressed by the junctional epithelium contributes to immune homeostasis by activating latent TGF-β and restraining excessive inflammation [53]. As inflammation progresses, leukocyte-associated β2 integrins, including LFA-1 and Mac-1, promote leukocyte adhesion, transmigration, and NF-κB-driven cytokine production, including IL-1β, TNF-α, and IL-6 [27,29]. These inflammatory mediators can subsequently alter β1 integrin-dependent adhesion, mechanotransduction, and ECM remodeling in PDL fibroblasts and other stromal cells.

Persistent inflammatory stimulation may also increase α9β1-associated signaling, which could contribute to MAPK-mediated inflammatory persistence through ERK, JNK, and p38 pathways [63]. However, its effects on leukocyte survival and retention in periodontitis remain incompletely established and are supported partly by evidence from related inflammatory models. The resulting inflammatory microenvironment may further influence αv-containing integrins. Reduced αvβ6-mediated TGF-β activation may weaken regulatory control, whereas αvβ3 engagement on osteoclasts cooperates with RANKL signaling to promote cytoskeletal organization, actin-ring formation, osteoclast activation, and alveolar bone resorption [54].

Meanwhile, β1 integrins, including α11β1, α2β1, and α5β1, regulate PDL fibroblast function through FAK- and RhoA/ROCK-associated pathways, thereby supporting matrix organization, mechanotransduction, and regenerative responses [20,22]. Disruption of these stromal pathways may modify ECM composition and mechanical cues, which can in turn affect leukocyte adhesion, migration, and activation. Crosstalk among β1-, β2-, and αv-containing integrins may therefore occur through shared signaling intermediates, including FAK/Src, PI3K/Akt, MAPK, NF-κB, and Rho-family GTPases, as well as through reciprocal communication between epithelial, immune, stromal, and osteoclast compartments.

Disease progression may reflect a shift from coordinated integrin signaling that maintains periodontal homeostasis toward a pro-inflammatory and pro-resorptive network characterized by persistent immune activation, altered stromal mechanotransduction, osteoclast-mediated bone destruction, and impaired tissue repair. However, many of these interactions remain incompletely defined in periodontal tissues and should be regarded as an integrated working model rather than as fully established pathways.

## 5. Integrin Signaling in the Transition from Periodontal Homeostasis to Disease

Periodontitis does not arise from abrupt structural failure but from a progressive shift in signaling balance within periodontal tissues [3,72]. Integrins play a pivotal role in this transition by integrating microbial challenge, immune activation, and mechanical stress into coordinated cellular responses [53,55,73]. The conversion from physiological regulation to destructive inflammation reflects qualitative and quantitative changes in integrin-mediated signaling networks.

### 5.1. Homeostatic State: Balanced Integrin Signaling

In health, integrin activity is tightly regulated. The β2 integrins facilitate controlled neutrophil trafficking into the gingival sulcus, ensuring microbial surveillance without excessive tissue damage [74]. The αvβ6 expressed in the junctional epithelium activates latent TGF-β, maintaining an anti-inflammatory microenvironment [52,53]—the β1 integrins in. PDL fibroblasts regulate ECM organization and mechanotransduction, supporting tissue stability under functional loading [15]. Osteoclast-associated αvβ3 operates within physiological limits to maintain balanced bone turnover [70]. In this state, integrin signaling is transient, spatially restricted, and counterbalanced by regulatory mechanisms.

### 5.2. Early Dysbiosis: Amplification of Immune-Integrin Signaling

The initial shift toward disease begins with microbial dysbiosis and persistent biofilm challenge. Increased bacterial burden promotes prolonged β2 integrin-dependent adhesion, recruitment, and signaling in neutrophils [75]. Prolonged integrin clustering enhances integrin-dependent signaling, amplifying pro-inflammatory cytokine production [76]. However, direct measurements of β2 integrin conformational activation in human periodontal lesions remain limited. Concurrently, microbial engagement of α5β1 and Mac-1 facilitates bacterial internalization and immune modulation, further intensifying inflammatory signaling [77]. At this stage, integrin-mediated immune responses begin to exceed regulatory capacity.

### 5.3. Breakdown of Regulatory Checkpoints

A critical transition point occurs with the downregulation of αvβ6 in the pocket epithelium. Reduced αvβ6 expression may limit the local capacity for latent TGF-β activation, thereby weakening an important anti-inflammatory checkpoint and allowing inflammatory signaling to persist. This loss of epithelial regulatory control shifts the local microenvironment toward chronic inflammation [78]. Increased α9β1 expression has been reported in inflamed periodontal tissues. Separately, functional studies from periodontal and related inflammatory models suggest that α9β1 engagement may promote MAPK-related signaling and support neutrophil survival. However, direct evidence linking α9β1 activation to prolonged neutrophil survival in human periodontal lesions remains limited, and increased tissue expression alone should not be interpreted as evidence of increased receptor activation [73].

### 5.4. Coupling of Inflammation to Bone Destruction

As inflammatory cytokines accumulate, RANKL expression increases in T cells, fibroblasts, and osteoblast-lineage cells. Osteoclast precursor differentiation is initiated through RANK signaling [79]. However, the transition to active bone resorption requires engagement of αvβ3 integrin. Engagement of αvβ3 with bone-matrix ligands activates c-Src- and Pyk2-dependent signaling, enabling cytoskeletal reorganization and formation of the resorptive apparatus. At this stage, integrin-mediated adhesion translates inflammatory signals into structural bone destruction [80].

### 5.5. Chronicity and Regenerative Failure

Persistent inflammation may disrupt β1 integrin-dependent adhesion and mechanotransduction in PDL fibroblasts and progenitor cells [35]. Disrupted integrin-mediated mechano-transduction impairs collagen organization and osteogenic differentiation. The tissue microenvironment becomes dominated by inflammatory integrin signaling rather than regenerative signaling [15,81]. Impaired efferocytosis, potentially involving αvβ5, may further delay resolution of inflammation.

### 5.6. Conceptual Model of Disease Transition

The progression of periodontitis can be interpreted conceptually as a phase-dependent reprogramming of integrin signaling from a balanced regulatory network to a dysregulated inflammatory and resorptive state. This proposed sequence comprises overlapping molecular and pathobiological processes and is not intended to correspond directly to the clinical stages or grades of periodontitis.

Under homeostatic conditions, β2 integrins, including αLβ2 and αMβ2, αvβ6, and β1 integrins, including α2β1 and α11β1, operate in a coordinated manner to maintain epithelial integrity, controlled immune surveillance, physiological bone turnover, ECM organization, and mechano-transduction. During early inflammation, prolonged β2 integrin-dependent adhesion and signaling, together with recruitment-associated pathways involving α4β1, enhance leukocyte infiltration, while α5β1 facilitates bacterial interaction and amplifies inflammatory signaling [37,55]. As disease progresses, reduced αvβ6 expression may diminish the epithelial capacity for TGF-β-mediated anti-inflammatory control. At the same time, upregulation of α9β1 promotes sustained MAPK signaling and prolongs inflammatory cell survival, marking a critical checkpoint failure [53]. During the bone-resorptive phase, αvβ3 integrin translates inflammatory signaling into structural damage by promoting RANKL-dependent osteoclast activation and alveolar bone resorption [54]. Concurrently, chronic inflammation disrupts β1 integrin-mediated mechano-transduction, impairing ECM organization and regenerative capacity, while dysfunction of resolution-associated integrins such as αvβ5 may further contribute to delayed tissue repair [35,36]. Although presented sequentially, these events are highly interconnected and may occur concurrently within the periodontal microenvironment. This integrin-centered model highlights that periodontitis is driven not only by microbial challenge but also by a progressive imbalance in integrin signaling, providing a mechanistic framework for identifying molecularly informed therapeutic targets.

## 6. Discussion

This review supports the concept that periodontitis is fundamentally a disease of dysregulated host signaling, wherein integrins act as central regulators of the transition from tissue homeostasis to chronic inflammation, structural destruction, and regenerative failure. Rather than functioning as passive adhesion receptors, integrins operate as dynamic signaling nodes whose activity evolves in response to changes in the periodontal microenvironment. The pathogenic importance of integrins, therefore, lies not only in the presence of specific subtypes but also in the temporal reprogramming of their signaling functions during disease progression [4,25].

During the proposed early molecular phase, integrin signaling is altered at the epithelial interface, where periodontal tissues first encounter dysbiosis biofilms. Under physiological conditions, epithelial integrins maintain barrier integrity and controlled attachment to the tooth surface and basement membrane. In particular, α3β1 and α6β4 support epithelial stability and attachment, while αvβ6 contributes to immune quiescence by activating latent TGF-β [6,24,25]. During the transition to early disease, microbial challenge disrupts this balance. Integrins such as α5β1 are increasingly relevant not only because they mediate matrix adhesion but also because they can be exploited by pathogens such as *Porphyromonas gingivalis* to facilitate internalization and the activation of downstream inflammatory signaling [20,35]. Thus, the earliest shift in integrin signaling reflects a transition from barrier maintenance toward host–pathogen interaction and epithelial vulnerability.

As inflammation develops, integrin signaling becomes increasingly dominated by leukocyte-associated pathways. β2 integrins, particularly LFA-1 and Mac-1, mediate leukocyte adhesion, transmigration, and activation [31,44]. In health, this signaling is transient and tightly regulated. In periodontitis, however, persistent microbial stimulation prolongs β2 integrin-dependent adhesion and signaling, shifting their function from controlled immune recruitment to sustained inflammatory amplification. The key pathogenic event is therefore not simply increased leukocyte trafficking, but failure to terminate β2 integrin-dependent signaling once host defense has been initiated. This results in continued production of reactive oxygen species, proteases, and pro-inflammatory cytokines, driving collateral connective tissue damage [39,46]. The severe periodontal destruction observed in leukocyte adhesion deficiency further emphasizes that β2 integrins represent a critical regulatory threshold between protective immunity and destructive dysregulation [33].

With progression to established inflammation, the integrin signaling network undergoes a further shift, characterized by the loss of regulatory control and the persistence of inflammatory signaling. A central event during the transition to persistent inflammation is the reduction in αvβ6-mediated regulation. Because αvβ6 normally activates TGF-β and restrains inflammatory escalation, its downregulation removes an important epithelial checkpoint, allowing inflammatory signaling to persist [6,24,25]. At the same time, α9β1 becomes more prominent in inflamed periodontal tissues. Functional and related inflammatory studies support its association with sustained MAPK signaling and prolonged neutrophil survival, although direct validation in human periodontitis remains limited [28,36]. Additional integrins, such as αDβ2, may contribute to macrophage retention, as suggested by evidence from broader inflammatory models, although this mechanism has not been directly established in periodontitis [55].

As the lesion becomes chronic, the signaling consequences extend beyond immune activation to affect stromal and matrix-associated functions. Collagen-binding integrins such as α2β1 and α11β1, which normally regulate ECM organization and mechano-transduction, become dysregulated during inflammation. This contributes to collagen breakdown and soft-tissue destruction while simultaneously impairing regenerative capacity [15,29,36,38]. Consequently, the periodontal microenvironment shifts from adaptive remodeling to one that is both inflamed and functionally compromised, reflecting a dual imbalance between persistent inflammatory signaling and loss of regenerative responsiveness.

The transition from soft tissue destruction to hard tissue loss is mediated most directly by αvβ3 integrin on osteoclasts. Although osteoclastogenesis is initiated by inflammatory mediators and RANKL, effective bone resorption requires αvβ3-dependent adhesion and cytoskeletal activation [42,44,70]. In this context, αvβ3 represents a critical phase-associated mediator that translates inflammatory signaling into irreversible structural damage. However, despite strong mechanistic evidence, the temporal relationship between αvβ3 activation and the severity of alveolar bone loss in human periodontal tissues remains incompletely defined, highlighting an important translational gap.

Another key feature of persistent destructive disease is failure of inflammatory resolution. In addition to persistent β2 and α9β1 signaling, impaired function of resolution-associated integrins, such as αvβ5, may delay the clearance of apoptotic cells and prolong inflammatory responses [82]. Although less well characterized than other integrin pathways, this deficit in resolution mechanisms likely contributes to persistent tissue breakdown and incomplete healing.

Up to this point, the progression of periodontitis can be interpreted as a sequential but overlapping evolution of integrin signaling. The early barrier-disruption phase may involve changes in the expression, localization, or organization of epithelial barrier-associated integrins (α3β1, α6β4) [16] and enhanced microbial interaction via α5β1 [17]. This is followed by prolonged β2-dependent immune activation, loss of αvβ6-mediated regulatory restraint, and α9β1-associated inflammatory persistence [63]. As inflammation becomes chronic, disruption of α2β1- and α11β1-associated matrix regulation may contribute to soft tissue destruction, although direct periodontitis-specific evidence remains limited [35], while αvβ3-dependent osteoclast activation drives bone resorption [13,30,54,70]. During failed-resolution and regenerative-impairment phases, impaired resolution mechanisms involving αvβ5 and dysfunctional β1 signaling lead to defective tissue repair [82]. Thus, periodontitis is best understood not as a static inflammatory condition, but as a dynamic process in which integrin signaling is progressively reprogrammed across epithelial, immune, stromal, and osteoclast compartments [6].

This progression-based perspective also provides insight into the heterogeneity of periodontal disease. Clinical outcomes may depend on the balance between destructive, regulatory, and reparative integrin pathways within the local microenvironment. Sites that retain αvβ6-mediated regulation and β1-dependent matrix function may remain stable. In contrast, those dominated by persistent β2 and α9β1 activation, together with strong αvβ3–RANKL coupling, are more likely to progress to severe tissue destruction [6,14,15,22,80].

Despite these insights, several important limitations remain. The available evidence is derived from in vitro experiments and animal models, while comprehensive in vivo data from human periodontal tissues remain limited. The strength and directness of evidence also vary considerably among integrin subtypes. Some mechanisms, including αvβ6-mediated TGF-β regulation and αvβ3-dependent osteoclast function, are supported by periodontal tissue, cellular, or animal studies. In contrast, other proposed roles, particularly α9β1-associated neutrophil survival, αDβ2-mediated macrophage retention, and αvβ5-related efferocytosis, are supported in part by the broader inflammatory literature and have not been fully validated in periodontitis. In addition, changes in integrin expression do not necessarily indicate altered conformational activation, ligand engagement, or downstream signaling. The temporal sequence of integrin-related changes, their interaction across epithelial, immune, stromal, and osteoclast compartments, and the point at which reversible inflammation becomes irreversible tissue destruction also remain incompletely defined. These limitations should be considered when interpreting the proposed framework, highlighting the need for longitudinal human studies and direct functional validation in periodontal tissues.

From a translational perspective, the proposed phase-dependent evolution of integrin signaling has important therapeutic implications. Early intervention may benefit from preserving epithelial barrier-associated and regulatory pathways, particularly αvβ6-mediated control [53]. Established inflammatory lesions may require selective modulation of β2- and α9β1-associated signaling to limit chronic immune activation [20,35]. When osteoclast-driven bone resorption and regenerative impairment predominate, suppression of αvβ3-dependent osteoclast activity and restoration of β1-mediated regenerative signaling may help prevent further tissue loss and improve healing [30,54]. These observations suggest that integrin-targeted therapies should be directed toward the dominant molecular imbalance within the periodontal microenvironment.

However, therapeutic modulation of integrins also carries important risks because these receptors contribute to normal immune surveillance, epithelial adhesion, ECM organization, angiogenesis, bone remodeling, and tissue repair. Inhibition of leukocyte-associated integrins may reduce inflammatory-cell recruitment but could also impair antimicrobial defense and increase susceptibility to infection. Suppression of epithelial or matrix-associated integrins may weaken barrier integrity, cell adhesion, wound healing, and periodontal regeneration. Likewise, inhibition of αvβ3 may reduce pathological osteoclast activity. Still, it may also interfere with physiological bone turnover and skeletal repair, whereas excessive stimulation of β1-integrin pathways could promote abnormal matrix remodeling or fibrosis. Additional concerns include integrin redundancy, shared ligands, compensatory signaling, and cell-type-specific effects, because the same integrin may exert protective and destructive functions in different tissue compartments. Therefore, future integrin-targeted strategies should prioritize subtype and cell-type selectivity, local rather than systemic delivery, limited treatment duration, and careful evaluation of infection risk, epithelial integrity, wound healing, and physiological bone remodeling.

In summary, integrin signaling in periodontitis progresses from homeostatic regulation to inflammatory amplification, checkpoint failure, osteoclast activation, and regenerative impairment. This conceptual framework links molecular signaling to periodontal disease progression and supports the development of molecularly informed host-modulatory strategies. However, clinical translation will require careful balancing of therapeutic efficacy against the essential physiological functions of integrins in host defense, tissue integrity, bone turnover, and regeneration.

## 7. Future Perspective

Future research should move beyond descriptive analyses of integrin expression and determine how integrin activation, ligand engagement, and downstream signaling change during periodontal disease progression. Although the proposed framework involves coordinated alterations across epithelial, immune, stromal, and osteoclast compartments, the temporal sequence and relative contributions of these alterations in human periodontal tissues remain poorly defined.

A major priority is the clinical validation of integrin–RANKL crosstalk, particularly the contribution of αvβ3 engagement to osteoclast activation and alveolar bone loss. Although experimental studies support this mechanism, longitudinal human evidence linking αvβ3 activity to disease progression remains limited. Similar studies are needed to determine how chronic inflammation affects β1 integrin-dependent mechanotransduction, how α9β1 contributes to inflammatory persistence, and whether restoration of αvβ6-mediated TGF-β regulation can promote inflammatory resolution.

Future studies should also distinguish changes in integrin abundance from conformational activation and functional signaling. This will require approaches that combine tissue localization, cell-surface expression, ligand-binding assays, activation-specific markers, and downstream signaling analysis. Spatial and single-cell methods may further clarify how integrin functions differ among epithelial cells, leukocytes, PDL cells, and osteoclasts within the same lesion.

From a therapeutic perspective, integrin modulation should prioritize subtype and cell-type selectivity, local delivery, and controlled treatment duration. Biomaterial-based delivery systems may help reduce systemic exposure. Still, candidate approaches must be evaluated for their effects on antimicrobial defense, epithelial integrity, wound healing, physiological bone remodeling, and regenerative capacity. Overall, future research should validate integrin signaling as a dynamic network of overlapping molecular processes before these pathways can be translated into clinically effective and safe therapies.

## 8. Conclusions

Integrin-mediated signaling links epithelial barrier function, immune cell recruitment, ECM regulation, osteoclast activity, and periodontal regeneration. During periodontitis, disruption of these coordinated functions may shift the periodontal microenvironment from homeostasis toward persistent inflammation, tissue destruction, bone resorption, and impaired repair.

The framework proposed in this review represents a conceptual sequence of overlapping molecular and pathobiological processes rather than a direct equivalent of the clinical stages or grades of periodontitis. The available evidence also varies substantially across integrin subtypes and encompasses changes in expression, ligand engagement, functional activity, and downstream signaling.

Integrins, therefore, represent important mechanistic regulators and potential therapeutic targets in periodontitis. However, clinical translation will require stronger human evidence and careful consideration of their essential physiological roles in host defense, epithelial integrity, bone turnover, and tissue regeneration.

## Data Availability

No new data were created or analyzed in this study.

## Abbreviations

The following abbreviations are used in this manuscript:

ECM	Extracellular matrix
FAK	Focal adhesion kinase
JNK	c-Jun N-terminal kinase
LFA-1	Lymphocyte function-associated antigen-1
Mac-1	Macrophage-1 antigen
MAPK	Mitogen-activated protein kinase
NF-κB	Nuclear factor kappa B
NFATc1	Nuclear factor of activated T cells cytoplasmic 1
PDL	Periodontal ligament
PI3K	Phosphoinositide 3-kinase
RANK	Receptor activator of nuclear factor kappa B
RANKL	Receptor activator of nuclear factor kappa B ligand
ROCK	Rho-associated protein kinase
Src	Src family kinases
TGF-β	Transforming growth factor beta
Pyk2	Proline-rich tyrosine kinase 2
IL-1β	Interleukin 1 beta
IL-6	Interleukin 6
TNF-α	Tumor necrosis factor alpha
Rho GTPases	Ras homolog family of small GTP-binding proteins
RhoA	Ras homolog family member A
